# Enhanced Efficiency and Reliability of AlGaN UVC-LED with Tapered Hole Injection Layer

**DOI:** 10.3390/mi16121376

**Published:** 2025-12-02

**Authors:** Linlin Xu, Yang Peng, Feng Wu, Wei Guo, Jiangnan Dai, Changqing Chen

**Affiliations:** 1 Wuhan National Laboratory for Optoelectronics, Huazhong University of Science and Technology, Wuhan 430074, China; linlinxu@hust.edu.cn (L.X.); wufeng123@hust.edu.cn (F.W.); daijiangnan@hust.edu.cn (J.D.); 2 School of Aerospace Engineering, Huazhong University of Science and Technology, Wuhan 430074, China; ypeng@hust.edu.cn; 3 Ningbo Institute of Materials Technology and Engineering, Chinese Academy of Sciences, Ningbo 315000, China

**Keywords:** AlGaN, UVC-LED, hole injection, reliability

## Abstract

In this work, the electrical and optical performance of AlGaN-based ultraviolet-C light-emitting diodes (UVC-LEDs) with a tapered Al-content hole injection layer was investigated both theoretically and experimentally. A total of 1000 h of real-time electrical stress was conducted to study the degradation process of such devices. UVC-LED incorporating a hole injection layer with a larger gradient was found to significantly suppress the degradation process compared to a sample with a smaller tapering gradient. Marginal efficiency droop of only 4.55% as well as 66% improved light output power, were identified for the proposed design under a current density of approximately 100 A/cm^2^. It was unambiguously demonstrated that UVC-LED with a greatly tapered hole injection layer facilitates both electron blocking and hole injection, providing a promising pathway towards the development of high-efficiency UV emitters.

## 1. Introduction

Aluminum Gallium Nitride (AlGaN) alloys, with their tunable wide direct bandgaps, high thermal and chemical stability, as well as intrinsic solar-blind ultraviolet response, offer significant potential for high-performance optoelectronic and electronic devices, including light-emitting diodes (LEDs), photodetectors (PDs), and high electron mobility transistors (HEMTs) [1,2,3,4,5]. When it comes to LEDs, AlGaN-based UVC-LEDs with emission wavelength below 280 nm are promising alternatives to traditional mercury lamp UV sources, exhibiting numerous advantages such as compact size, energy efficient, long-term reliability, and absence of toxic mercury element, thus are ideal candidates for a wide range of applications, including water purification, medical disinfection, and air sterilization [6,7,8]. Despite their benefits, UVC-LEDs are facing a long-standing challenge of unsatisfactory external quantum efficiency (EQE) below 10% [9,10,11]. Several issues are responsible for the low EQE value of UVC-LED, including inferior crystal quality that leads to low radiative recombination rate, poor light extraction efficiency due to enhanced TM light polarization, and high p-doping activation energy due to low hole concentration, etc. [12,13]. Among these challenges, insufficient hole injection due to low carrier concentration in the p-type region, as well as a high potential barrier for holes at EBL/p-AlGaN interface, significantly limits the quantum efficiency and impacts the overall performance and reliability of UVC-LEDs. Numerous efforts have been made to enhance the hole injection by optimizing the design of the active region [14,15], last quantum barrier [16], electron blocking layer (EBL) [17,18], current spreading layer [19], p-AlGaN layer [20], and p-GaN layer [21,22] of the UVC-LEDs. Zhang et al. proposed a very thin Al_0.5_Ga_0.5_N layer in p-EBL to achieve a high local hole concentration and promote intra-band tunneling, effectively reducing the effective barrier height for holes [23]. Yin et al. proposed an Al-content engineered superlattice EBL in order to improve kinetic energy during hole transport, and superior electron blocking and hole injection were obtained [24]. For the practical application of UVC-LEDs, the optical and electrical performances as well as reliability are all crucial. A great number of studies were focused on structural optimization of UVC-LED, but only a few of them have also conducted electrical stress characterization to investigate the long-term performance and stability of the devices [25,26].

In this study, a group of UVC-LEDs with different tapered hole injection layer (HIL) designs were designed and fabricated. For the electrical stress investigation, the UVC-LEDs were lit up for up to 1000 h under a constant current density of 34 A/cm^2^. Optical and electrical properties of the devices were comprehensively compared to reveal the influence of the Al-content gradient in HIL on the light output power and efficiency droop of UVC-LEDs. Meanwhile, numerical simulations were also conducted to uncover the underlying mechanism that contributes to the performance variation.

## 2. Experiment Details

### 2.1. Device Fabrication

**Figure 1:**
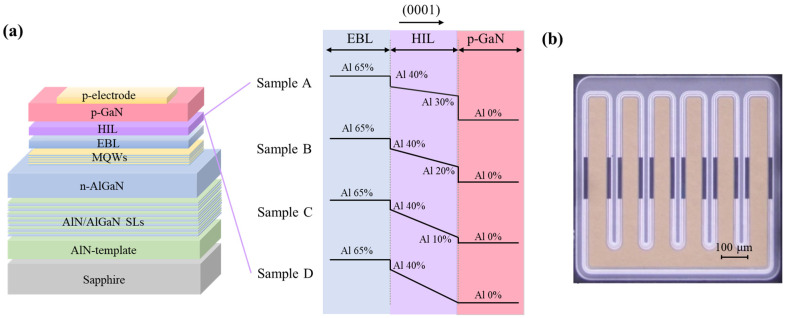
(**a**) Schematic diagram of the UVC-LEDs with different tapered HIL, (**b**) the optical microscope image of the UVC-LED chip under evaluation.

A group of UVC-LEDs with a designed emission wavelength of 280 nm were grown on (0001) sapphire substrates using metalorganic chemical vapor deposition (MOCVD). The heterostructures are shown in Figure 1a, which consists of a 3 μm thick AlN buffer layer, 20 periods of AlN/AlGaN superlattices (SLs), a 3 μm thick Si-doped n-AlGaN contact layer with a Si doping concentration of 1 × 10^19^ cm^−3^, 5 periods of undoped Al_0.48_Ga_0.52_N/Al_0.55_Ga_0.45_N (3 nm/10 nm) multi-quantum wells (MQWs), a 50 nm Mg-doped p-Al_0.65_Ga_0.35_N EBL, a 20 nm HIL with gradually degrading Al content, and a 200 nm Mg-doped p-GaN contact layer. The Mg doping concentrations in the p-EBL, p-HIL, and p-GaN contact layer are 1 × 10^19^ cm^−3^, 1 × 10^18^ cm^−3^, and 5 × 10^18^ cm^−3^, respectively. The only difference among these devices is the gradient of Al content in HIL, where the Al-content differences between the top and bottom of the HIL are 10%, 20%, 30% and 40% for devices A, B, C, and D, respectively, as shown on the right of Figure 1a. After MOCVD epitaxy, all wafers underwent a standard flip-chip device fabrication process with a chip size of 0.762 × 0.762 mm^2^, or 30 × 30 mil^2^. The optical microscope image of the UVC-LED chip under evaluation is shown in Figure 1b. The active area of the UVC-LED is 0.294 mm^2^ after mesa etching. An interdigital electrode structure was applied to the chips, and annealed Ti/Al/Ti/Au and Ni/Au metals were used as n-contact and p-contact metals. The detailed growth and chip fabrication conditions can be found in our previous works [27].

### 2.2. Characterization

Electroluminescence (EL) spectra, current–voltage characteristics, and the light output power were achieved by a customized Everfine ATA-1000 photoelectric analysis system with a 30 cm diameter integrating sphere at room temperature. Current stress was conducted in constant-current mode, and the devices were mounted on a large heatsink and tested in an air-conditioned cleanroom laboratory under controlled ambient temperature to minimize the thermal effects of the devices. The Keithley 2400C source measure unit was used to provide current bias during current stress. Light output powers and operation voltages after a certain period of aging times were achieved by the Everfine ATA-1000 system.

### 2.3. Numerical Investigation

Numerical investigation was conducted by commercial software APSYS 2009 via Crosslight Inc. (Vancouver, BC, Canada). It is capable of solving the Schrödinger equation, Poisson’s equation, and the current continuity equation, so as to investigate the carrier distributions and energy bands of the wurtzite semiconductor devices [28]. Considering the nonradiative recombination process is involved in the active region, which may lead to carrier loss, the Auger recombination coefficient and Shockley–Read–Hall recombination lifetime are set as 1 × 10^−30^ cm^6^/s and 1.5 ns, respectively. The conduction and valence band offsets are assumed to be 0.7/0.3 for AlGaN materials. The Mg dopant ionization efficiency is assumed to be 1%, and the operating temperature is set to be 300 K. Detailed parameters used in the simulation can be found elsewhere [29].

## 3. Results and Discussions

**Figure 2:**
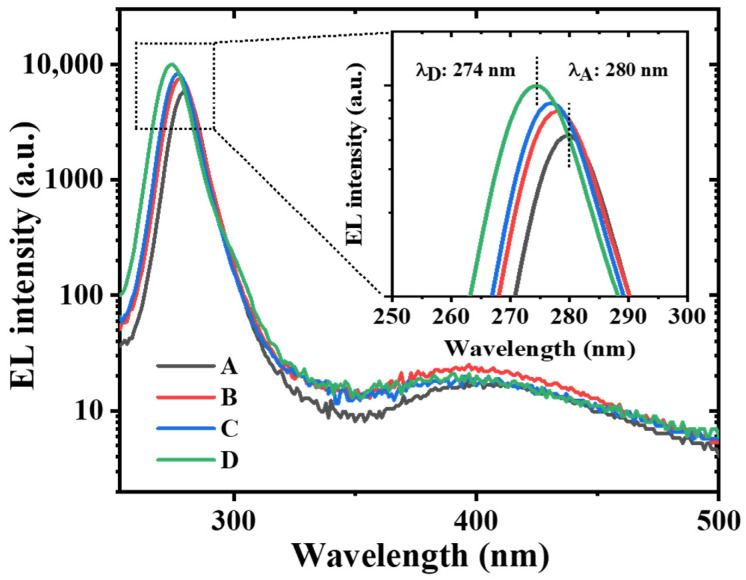
EL spectra of the devices measured at a current level of 100 mA before electrical stress. The inset shows the monotonous blue-shift of the main peak from 280 nm to 274 nm for device A to device D.

The EL spectra of the UVC-LEDs under 100 mA injection current are shown in Figure 2. The main emission peaks of device A to device D are 280 nm, 278 nm, 277 nm, and 274 nm, respectively, revealing a blue-shift trend. A possible explanation for this trend is the enhancement of hole injection and improved electron confinement thanks to the introduction of tapered HIL, which will be further discussed below. A more tapered HIL introduces a stronger electrical field with a higher density of polarization-induced hole concentration, as well as improving the overlap of electron and hole wave functions due to the carrier screening effect. Therefore, it can alleviate the quantum-confined Stark effect, consistent with the blue-shift of the emission peak. Meanwhile, the intensity of parasitic defect luminescence at longer wavelengths is more than two orders of magnitude weaker than the main peaks, which may be derived from the p-type AlGaN layer.

**Figure 3:**
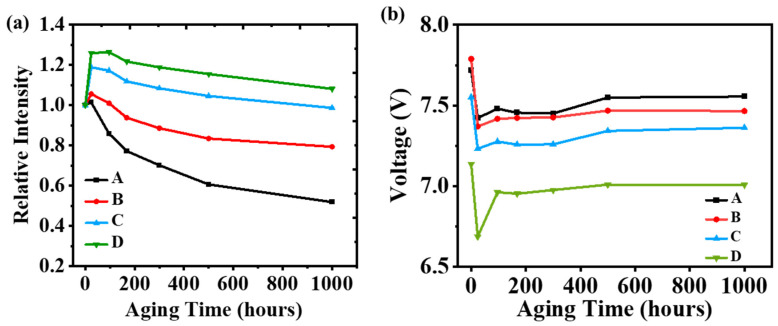
(**a**) Normalized LOP intensity of the UVC-LEDs as a function of aging time, (**b**) operation voltage in terms of aging time.

The normalized light output power (LOP) over electrical stress aging time up to 1000 h under constant current density of 34 A/cm^2^ is shown in Figure 3a. LOP value declines with increasing aging time for device A. However, there is an enhancement of LOP within the first 24 h for devices B, C, and D, and the enhancement factor significantly increases from B to D. Furthermore, the degradation rates of devices B to D are slower than that of device A. The trend of the LOP value can be explained as follows. On one hand, electrical stress can activate the Mg dopant due to the local annealing effect at the beginning of the aging process, leading to higher hole concentration with improved p-type conductivity. On the other hand, electrical stress can also generate defects in the active region with an increased amount of nonradiative recombination centers [30]. It is believed that the combination of these two factors results in the “roll-over” behavior of the time-dependent LOP curve. The former mechanism dominates the first aging stage as the LOP of all the UVC-LEDs shows unambiguous peak values at about 24 h. Subsequently, LOP starts to decline after 24 h of aging. The tradeoff between these two mechanisms leads to the final LOP profile. Specifically, with respect to devices A and B, the LOP declined dramatically after the peak value. It indicates that the latter mechanism becomes dominant shortly after the peak at 24 h. However, for devices C and D, even after a current stress for 1000 h, the LOP is still larger than the initial value. It manifests that the defect accumulation process is decelerated in the active region of these UVC-LEDs, especially in device D. Such dopant activation and defect generation processes are crucial in the development of III-nitride light emitters.

The variation of forward voltage as a function of aging time is shown in Figure 3b. It is revealed that the forward voltage significantly drops at the beginning of the aging process, then gradually increases over time. The drop of forward voltage is synchronized with the enhanced LOP as shown in Figure 3a, which is ascribed to the enhanced hole activation rate herein, resulting in lower on-state resistance. According to the aforementioned analysis, the p-type annealing effect dominates the aging process in the first place. With the process of Mg dopant activation in the p-type layer, the accumulated holes may result in lower resistance and forward voltage. However, the forward voltage is dependent on the tradeoff between the improvement of hole injection and reduced radiative recombination due to the generated defect, as mentioned above. Over the aging time from 24 h to 1000 h, the forward voltage gradually increases, suggesting an absence of a prominent current leakage path during aging. So that the increase in forward voltage is supposed to be mainly caused by the increase in the resistivity of the contacts [31]. Among different designs of tapered HIL, device D with the largest Al-content slope introduces the strongest electrical field, may effectively enhance the hole concentration and reduce the barrier for holes, thus exhibiting the lowest forward voltage and highest LOP.

**Figure 4:**
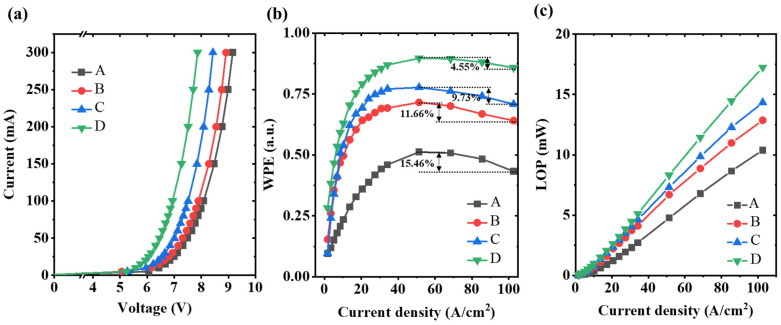
(**a**) Current–voltage characteristics of the UVC-LEDs, (**b**) WPE, and (**c**) LOP in terms of current density after 1000-h electrical stress.

The current–voltage characteristics of the four UVC-LEDs after 1000-h electrical stress are shown in Figure 4a. Device D shows the lowest forward turn-on voltage compared to other samples. Since the electrode contacts are the same for all four samples, the increase in turn-on voltage vs. aging time can only be attributed to the different potential barriers at the EBL/HIL/p-GaN interface, as will be discussed later. Under the same current level, lower forward voltage and higher LOP refer to higher wall plug efficiency (WPE). WPE is a production of injection efficiency, radiative efficiency, light extraction efficiency, and electrical efficiency. The normalized WPE curves in terms of current density from 0 to 100 A/cm^2^ are shown in Figure 4b, and the efficiency droop profile is revealed for all devices. According to the ABC model for carrier recombination processes, the nonradiative recombination processes inside the quantum wells can either be defect-related Shockley–Read–Hall recombination or Auger recombination [32]. The efficiency droops are 15.46%, 11.66%, 9.73% and 4.55% for devices A to D, respectively. The suppression of efficiency droop clearly manifests the best electron blocking capability in device D. The LOP versus current density is plotted in Figure 4c, showing that the LOP of device D reaches 17 mW at a current density of around 100 A/cm^2^, 66% higher than that of device A.

**Figure 5:**
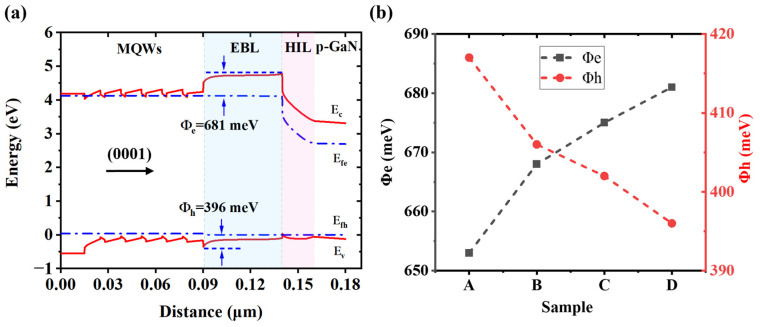
(**a**) Simulated energy bands of device D, and (**b**) summary of Φe and Φh of the four devices.

To throw light upon the origin of the promoted current injection and LOP, a numerical investigation is conducted. The energy bands in the MQW region are almost the same due to identical growth conditions. However, the band profiles of the EBL and HIL are distinct owing to different designs of the tapered HIL. Typically, the simulated energy bands of device D are presented in Figure 5a. E_c_, E_fe_, E_fh,_ and E_v_ are denoted as the conduction band, the quasi-Fermi level for electrons, the quasi-Fermi level for holes, and the valence band, respectively. Φ_e_ and Φ_h_ denote the effective potential barrier height of electrons and holes. As shown in Figure 5b, the value of Φ_e_ is calculated to be 653 meV, 668 meV, 675 meV, and 681 meV for device A to device D, respectively. Meanwhile, the values of Φ_h_ decrease from device A to device D, which are calculated to be 417 meV, 406 meV, 402 meV, and 396 meV for device A to device D, respectively. Demonstrating that in device D, the hole injection across the EBL to the active region of MQWs encounters a lower potential barrier at the interface between EBL and HIL, which is facilitated by the greatly tapered HIL design. The calculated effective potential barriers demonstrate an improvement in both electron blocking and hole injection from device A to D. Consequently, the variation in electron and hole potential barriers among these four samples is the root cause for the performance difference.

**Figure 6:**
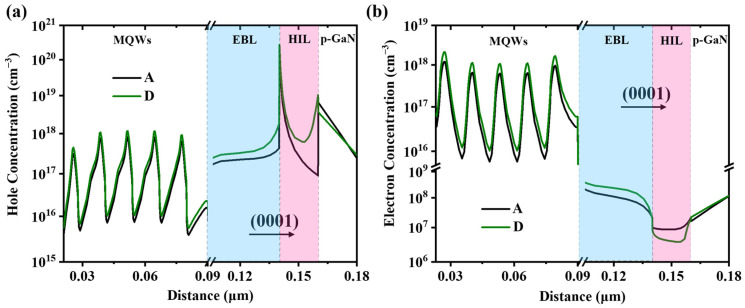
(**a**) Hole and (**b**) electron concentrations of the MQWs, EBL, HIL, and the p-GaN region for devices A and D.

To better interpret the underlying carrier distribution of the devices, the hole and electron concentrations in the MQWs, EBL, HIL, and the p-GaN region are also investigated by APSYS. The carrier concentrations of device A and D are presented in Figure 6 for comparison due to the largest Al-content difference of the HIL. Although the structure of MQWs is identical, device D with higher Φ_e_ and lower Φ_h_ has better electron blocking and hole injecting performance, leading to the improvement of both carrier concentrations in the active region. With respect to the HIL region, the hole and electron distributions in both devices are in accordance with their effective potential barriers. Particularly, device D exhibits the highest hole concentration and lowest electron concentration in HIL, representing the improvement of hole injection and suppression of electron leakage. It is known that the electron leakage level accounts for the efficiency droop for UVC-LEDs. According to the abovementioned analysis, device D with the lowest efficiency droop also shows the best electron blocking performance, which demonstrates that the simulated results agree well with the experimental data.

## 4. Conclusions

In summary, we have designed and fabricated four UVC-LEDs with tapered HIL structures of different Al-content gradients. Both the experiment and simulation outcomes demonstrate that the device with a greatly tapered HIL shows the smallest efficiency droop, the highest LOP, and the smallest operation voltage. Simulations were conducted to reveal the carrier distributions and energy bands, establishing a strong correlation between device performance and potential barriers of carriers. The results of this work are beneficial for the development of high-efficiency AlGaN-based UVC-LEDs.

## Data Availability

Data will be made available on request.
